# Gut Microbiome Modulation and Faecal Microbiota Transplantation Following Allogenic Hematopoietic Stem Cell Transplantation

**DOI:** 10.3390/cancers13184665

**Published:** 2021-09-17

**Authors:** Karolina Kaźmierczak-Siedlecka, Karolina Skonieczna-Żydecka, Jarosław Biliński, Giandomenico Roviello, Luigi Francesco Iannone, Alessandro Atzeni, Bartosz Kamil Sobocki, Karol Połom

**Affiliations:** 1 Department of Surgical Oncology, Medical University of Gdansk, 80-214 Gdańsk, Poland; polom.karol@gumed.edu.pl; 2 Department of Biochemical Sciences, Pomeranian Medical University in Szczecin, Broniewskiego 24, 71-460 Szczecin, Poland; karzyd@pum.edu.pl; 3 Department of Hematology, Transplantology and Internal Medicine, Medical University of Warsaw, 02-097 Warszawa, Poland; j.bilinski@wum.edu.pl; 4 Department of Health Sciences, Section of Clinical Pharmacology and Oncology, University of Florence, 50139 Florence, Italy; giandomenico.roviello@unifi.it; 5 Department of Health Science, University “Magna Graecia” of Catanzaro, 88100 Catanzaro, Italy; iannone@unicz.it; 6 Human Nutrition Unit, Department of Biochemistry and Biotechnology, Rovira i Virgili University, Faculty of Medicine and Health Sciences, Campus Vapor Vell, 43210 Reus, Spain; alessandro.atzeni@urv.cat; 7 International Research Agenda 3P—Medicine Laboratory, Medical University of Gdansk, 80-214 Gdańsk, Poland; b.sobocki@gumed.edu.pl

**Keywords:** allogenic hematopoietic stem cell transplantation, fecal microbiota transplantation, prebiotics, probiotics, synbiotics, postbiotics, intestinal integrity, gut microbiota

## Abstract

**Simple Summary:**

Allogenic hematopoietic stem cell transplantation (allo-HSCT) represents a significant part of the treatment for hematologic malignancies and other types of diseases. Allo-HSCT-related complications, such as conditioning chemotherapy, graft-versus-host disease, mucositis, irradiation, administration of antibiotics, recurrent *Clostridioides difficile* infection, and many others, can alter the gut microbiota composition, leading to its imbalance. Consequently, the disruption of gut microbiota homeostasis and loss of gut-barrier integrity affect therapy efficacy. Thus, restoring gut microbiota diversity and maintaining its balance seem to be strongly needed in these cases. Promising effects were observed after fecal microbiota transplantation (FMT). Notwithstanding, FMT efficacy was confirmed in *Clostridium difficile* infection treatment in HSCT recipients. Gut microbiota may be also modified by prebiotics, probiotics, synbiotics, and postbiotics. However, the administration of fungal probiotics is associated with the risk of fungemia/septicemia, especially in immunocompromised patients.

**Abstract:**

Nowadays, allogenic hematopoietic stem cell transplantation (allo-HSCT) is a curative therapy that is mainly recommended for hematologic malignancies. However, complications (such as graft-versus-host disease, mucositis, disease relapse, and infections) associated with the HSCT procedure contribute to the development of gut microbiota imbalance, gut-barrier disruption, and increased intestinal permeability. In the present narrative review, the crosstalk between gut microbiota products and intestinal homeostasis is discussed. Notably, gut-microbiota-related aspects have an impact on patients’ clinical outcomes and overall survival. In accordance with the most recent published data, gut microbiota is crucial for the treatment effectiveness of many diseases, not only gastrointestinal cancers but also hematologic malignancies. Therefore, it is necessary to indicate a therapeutic method allowing to modulate gut microbiota in HSCT recipients. Currently, fecal microbiota transplantation (FMT) is the most innovative method used to alter/restore gut microbiota composition, as well as modulate its activity. Despite the fact that some previous data have shown promising results, the knowledge regarding FMT in HSCT is still strongly limited, except for the treatment of *Clostridium difficile* infection. Additionally, administration of prebiotics, probiotics, synbiotics, and postbiotics can also modify gut microbiota; however, this strategy should be considered carefully due to the high risk of fungemia/septicemia (especially in case of fungal probiotics).

## 1. Introduction

The gut microbiota is a complex ecosystem consisting of bacteria, fungi, viruses, and Archaea [1,2] that influences multiple physiological functions, including metabolism, inflammation, and immunity’ response [3]. The clinical significative role of the gut microbiota in the development and management of several diseases has been highlighted. Indeed, increasing evidence revealed that an altered gut microbiota might be involved in the pathophysiology of the gastrointestinal tract and systemic diseases (e.g., neuropsychiatric and cardiovascular disorders), as well as in the development and maintenance of several malignancies [4]. Preclinical data suggest that the microbiota modulation could become a novel strategy for improving the efficacy of cancer therapies; however, just a few clinical studies have been performed so far, and a translation from basic science is strongly needed [5]. Currently, fecal microbiota transplantation (FMT) and its variations (e.g., washed microbiota transplantation, bacterial consortia transplantation, and live biotherapeutics) are the most innovative method used to markedly modify the gut microbiota composition and consequently its function [1,6,7,8]. 

Nowadays, FMT has been approved by the Food and Drug Administration (FDA) as clinical treatment for recurrent *Clostridioides difficile* infection (RCDI) in 2013 [9,10]. FMT is classified as a drug in the USA and Canada. However, in Europe, there is not a precise described recommendation to classify it [10]. 

FMT is the most effective treatment for RCDI, with an estimated efficiency around 60–90% as a single therapy [11]. According to recent studies, FMT may be also effective in reducing gut-multidrug-resistant bacteria (MDRB) [12]. The potential benefits of FMT for patients with hematologic malignancies and underwent allogeneic hematopoietic stem cell transplantation can be multiple, including direct effects, due to replacement of the gut microbiota from healthy donors, and indirect effects (e.g., restoration of epithelial cells integrity and regulation of short-chain fatty acids (SCFAs) production) [13]. 

Herein, we reviewed the potential roles of FMT in the treatment of hematologic malignancies and allogeneic hematopoietic stem cell transplantation, as well as their related complications (treatment of MDRB gut colonization, RCDI, graft-versus-host disease, immunotherapy, infectious complications, etc.) and the use of microbiota supplementations, such as prebiotics, probiotics, synbiotics, and postbiotics. 

## 2. Gut-Microbiota-Related Aspects 

### 2.1. Gut Microbiota in Hematologic Malignancies and Allogeneic Hematopoietic Stem Cells Transplantation 

Preclinical studies with animal models seem to demonstrate an active and bidirectional interplay between the gut microbiota and the immune system. Indeed, the development of some lymphomas, such as mucosal-associated lymphoid tissue (MALT) lymphoma, might be linked to the presence of gut detrimental bacteria [14]. Further animal studies have evidenced that specific bacteria are able to promote the differentiation of pro-inflammatory cells colonizing the gut and migrating to the bone marrow in transgenic mice, favoring the progression of multiple myeloma (MM) [15]. Indeed, a reduced diversity in the bacterial community and the enrichment in species correlated with unhealthy status was described in MM patients [16]. Gut microbiota impacting the degree of antigen stimulation of plasma cells in MM might have a role in disease progression. Furthermore, multiple myeloma therapies are frequently associated with gastrointestinal adverse events [17]. The role of the gut microbiota and alterations of its metabolic functions in the development of MM were also recently explored. The analysis of the bacterial community revealed a significant enrichment of nitrogen-recycling bacteria in MM patients, also linked to interactions with the host metabolome. These bacteria blooming most likely result from the regulation of urea nitrogen accumulated during MM progression. The FMT procedure in mice showed an interaction between MM-enriched bacteria and MM progression through urea nitrogen recycling [18]. Finally, other studies demonstrated that enriched species also promoted MM progression via de novo synthesis of glutamine in mice [18]. 

The study of the gut microbiota can be informative for the improvement of therapeutic strategies aiming to ameliorate the adverse outcomes of post-immunotherapy infections occurring in hematologic malignancies [19]. A study conducted in children with acute lymphoblastic leukemia (ALL) showed that the gut microbial profile might serve as a valid predictive biomarker of infection during chemotherapy [20]. Accordingly, improvements in the prevention and the prognosis of childhood leukemia based on promoting a heathy gut microbiota are currently explored [21]. The changes of gut microbiota in children with ALL were also examined during different timepoints of the chemotherapy, showing small differences between ALL patients and healthy controls even after cessation of chemotherapy. These changes may be beneficial in childhood cancer survivors but is still not clear the impact in subsequent health perturbations in the same patients [22]. In a multicenter study it was observed that gut decontamination therapy contributes to lower incidence of acute gastrointestinal GvHD in children undergoing allo-HCT [23]. Alterations of gut microbiota in children with aGvHD, regarding a significant reduction of commensal anaerobes (mainly *Faecalibacterium prausnitzii*—beneficial, butyrate-producing bacterium) and an increase of opportunistic bacteria are observed [24,25,26]. Additionally, Biagi et al. reported that pediatric patients who developed gut aGvHD presented gut microbiota dysbiotic changes before HSCT [27]. The results of Simms-Waldrip et al.’s study suggests that anti-inflammatory *Clostridia* depletion in the gut microbiota induced by antibiotics is related to the development of GvHD in pediatric patients, thus it has also an impact on clinical outcome [26]. Moreover, in another study, it was shown that pediatric patients undergoing HSCT and receiving antibiotics therapy against anaerobic microorganisms present a lower level of short-chain fatty acids—butyrate and propionate [28]. Overall, aforementioned studies have shown that loss of bacterial diversity and increased use of antibiotics are related to GvHD. 

Notably, allo-HSCT is a curative therapy indicated for mainly hematologic malignancies. Notwithstanding, this procedure can be associated with complications, such as graft-versus-host disease (GvHD), disease relapse, and infections [19]. On the other hand, HSCT has also been related to alterations in the gut microbiota composition. Conditioning chemotherapy, irradiation, administration of antibiotics, GvHD, mucositis, and RCDI are the main factors contributing to microbiota alterations, which may occur after allo-HSCT procedure [19,29]. Taur et al.’s study showed that the diversity and stability of gut microbiota balance were disrupted during allo-HSCT [30]. An increased abundance of bacteria belonging mainly to the *Enterococcus* and *Streptococcus*, as along with *Proteobacteria* phylum was observed. Indeed, *Enterococci* was increased three-fold by metronidazole, whereas *Proteobacteria* was reduced 10-fold by fluoroquinolone administration. Noteworthy, a nine-fold and five-fold increase in the risk of bacteremia by vancomycin resistant *Enterococcus* and proteobacterial abundance were evidenced respectively [30]. Another study also reported an increase in the *Enterococcus* genus during chemotherapy treatment in patients with acute myeloid leukaemia [21]. Moreover, in a study comprising 64 patients, on day 12 after allo-HSCT, increased diversity of gut microbiota was associated with lower GvHD-related mortality, and the abundance of *Blautia* was related to reduced GvHD lethality and improved overall survival (OS) [31]. Recently, in 2020, Peled et al. reported that microbiota may be assessed as a predictor of mortality in allo-HSCT recipients [32]. In this study, the analysis of 8767 fecal samples from 1362 patients undergoing allo-HSCT, using 16S rRNA gene sequencing, was conducted. The authors noted that the higher diversity of gut microbiota was linked to lower risk of death [32]. 

On the other hand, low diversity of gut microbes has been linked to worsening clinical outcomes and increasing mortality after allo-HSCT [19]. Loss of gut microbiota diversity and overgrowth of opportunistic bacteria (*Enterococcus* genus and *Proteobacteria* phylum) were related to increased risk of infections incidence and mortality after allo-HSCT [33]. Additionally, in a study including 42 participants [28], a loss of intestinal commensals producing SCFAs was strongly associated to increased gut microbiota imbalance after HSCT, with lower levels of SCFAs in patients treated with antibiotics. Moreover, the amount of SCFAs (namely propionate and butyrate) was reduced in patients who develop GvHD [28]. 

Gut microbiota was associated with immune cell dynamics in humans, as shown in Schluter’s et al. trial [34]. This study regarded hundreds of hospitalized/closely monitored patients receiving HSCT. Notably, an analysis of daily changes in circulating neutrophil, monocyte, and lymphocyte counts and more than 10,000 longitudinal microbiota samples showed a consistent link between gut bacteria and dynamics of immune cells [34]. 

### 2.2. Intestinal Integrity: Crosstalk between Gut Microbiota Products and Intestinal Homeostasis 

Intestinal mucosa is a barrier which prevents the access of potentially harmful content of the intestinal lumen to the systemic circulation. Functional impairment of the intestinal barrier can be associated with many diseases as inflammatory bowel disease, celiac disease, irritable bowel syndrome, type I diabetes, multiple sclerosis, rheumatoid arthritis, autism spectrum disorders, and GvHD [35,36]. Fisher et al. indicated that chemotherapy or total body irradiation during allo-HCST can lead to intestinal epithelium damage and subsequently to the microbial translocation into sterile compartments, causing immune activation. Vancomycin-resistant *Enterococcus*, viridians-group *Streptococcus*, and aerobic Gram-negative bacteria are considered as the most common factors contributing to bloodstream infection after allo-HCST [37]. Bacterial components and endogenous signals which are released from damaged epithelium stimulate antigen-presenting cells producing proinflammatory cytokines (i.e., TNF-α, IL-1, IL-6, IL-10, IL-12, and TGF-β) and prime donor-derived T-cells. These interactions with the microbiome are connected with the stimulation of TLRs. The transfer of HoxB8 neutrophils without TLR-2, -3, -4 and -9 expression reduced GvHD severity compared to transfer of WT HoxB8 neutrophils (with expression of selected TLRs) which seems to indicate a role of TLR in promoting GvHD. Moreover, activation of alloreactive T cells caused by translocation process was associated with destroying host tissues, which is main cause of morbidity and mortality in GvHD [37,38]. Notably, pre-transplant conditioning may contribute to intestinal barrier disruption, as well as apoptosis of Paneth cells and enterocytes [39]. A gut microbiota with predominant *Enterococcus* genus was observed. Moreover, low concentrations of SCFAs and lactase, as well as a high level of lactose, are also noted. An increased MLCK (myosin light chain kinase) expression caused loose of tight junctions and PAMPs (pathogen-associated molecular patterns), such as lipopolysaccharide translocation into lamina propria. An overproduction of proinflammatory mediators (IFN-γ, TNF-α, and IL-5) was also observed [39]. Therefore, the intestinal barrier protection provided by therapeutic modifications of gut microbiota seems be to extremely significant in allo-HSCT recipients. 

Gut microbiota is an important factor in maintaining intestinal integrity and an appropriate microenvironment that prevents GvHD and other diseases. Considering its potential influence, the gut microbiota and its alterations should be kept in mind during treatment. The Xuebijing injection (XBJ), a China Food and Drug Administration-approved Chinese medicine injection, composed of extracts from five medicinal herbs, namely Honghua (*Carthamus tinctorius* flowers), Chishao (*Paeonia lactiflora* roots), Chuanxiong (*Ligusticum chuanxiong* rhizomes), Danggui (*Angelica sinensis* roots), and Danshen (*Salvia miltiorrhiza* roots), in combination with a reduced dose of cyclosporine A, resulted in a better option than cyclosporine alone in improving survival of mice with acute GvHD. The impact of this combination was associated with several effects as reduced IL-6 and IL-12 levels in peripheral blood, inhibition of *Enterococcus* and *Escherichia coli*, improved integrity and reduced permeability of intestinal tissue of mice with acute GvHD [40]. The microbiota composition was reversed at the phylum, genus and species level [40]. In another study, Routy et al. showed that the use of antibiotics targeting intestinal bacteria, significantly decreased the median overall survival and was connected with more severe acute GvHD [41]. Shono et al. also confirmed that antibiotics use, especially piperacillin–tazobactam, was associated with increased gut microbiota compositional perturbation. In addition, imipenem–cilastatin treatment in mice caused loss of the protective layer of mucus in the colon and intestinal barrier function impairment [42]. Moreover, the study from Liu et al. proved that the administration of valproic acid (VPA) changed gut microbial composition and metabolites produced by the gut microbiota [43], whereas another study suggests that VPA reduces GvHD severity and mortality. This mechanism is based on VPA histone deacetylase inhibitor activity that implies donor CD4+ T cells reduction [44]. There is a lack of studies directly describing associations among microbiota, VPA and allo-HSCT and this topic should be considered as a promising target for future studies. Investigating the influence of treatments on microbiota is a promising direction to prevent GvHD. Microbe-derived products are the next essential factor which play a role in intestinal barrier maintenance and have association with immune activation in GvHD. Indeed, higher circulating concentration of SCFAs (i.e., burytate and prioponate) in two independent patient cohorts was associated with a lower risk of GvHD incidence. Moreover, another study conducted by Fujiwara et al. indicated that protective effect of SCFAs require GPR43-mediated ERK phosphorylation and activation of NLPR3 inflammasome in non-hematopoietic host’s tissues. GPR43 is expressed in cells contributing to GvHD development such as: antigen-presenting cells, donor T cells and intestinal epithelial cells [45]. In addition, Mathewson et al. investigated that SLC5A8 (butyrate monocarboxylate transporter) was decreased in intestinal epithelium and its decrease may lead to reducing butyrate intake in a feedback mechanism. Another interesting mechanism was that butyrate changed the ratio anti-apoptotic to pro-apoptotic cells, causing an increase in the expression of the proteins contributing to junctional integrity [46]. 

As mentioned above, the interaction between gut microbiota, as well as its products, and immune system was observed. Among others, gut microbiota is controlled by Paneth cells, secretory goblet cells, and intestinal epithelial cells [47]. Gut microbiota affects immune system both locally and systemically [48]. Most commonly used probiotics, such as *Lactobacillus* spp. and *Bifidobacterium* spp., can modify gut microbiota and have an impact on gastrointestinal immunity. Moreover, they regulate T and B cells production, maintain Th1/Th2 balance, increase the levels of IgA, and stimulate the secretion of anti-inflammatory mediators [48]. Therefore, the modulation of gut microbiota through therapeutic strategies (not only administration of probiotic strains) seem to be beneficial also in the context of immune system enhancement [47,48]. 

## 3. Fecal Microbiota Transplantation in Allogeneic Hematopoietic Stem Cell Transplantation 

### 3.1. Definition, Preparation, and Implication 

An ancient method of FMT was introduced approximately 1700 years ago by a Chinese medical scientist and consisted in a mixture called “yellow soup”, administered in case of severe food poisoning or diarrhea [49]. Nowadays, FMT has become a modern, evidence-based therapeutic method which profoundly alter the gut microbiota [1]. Nevertheless, choosing the most appropriate donor, the dosage, and the optimal method for FMT administration is still strongly limited [50]. FMT is defined as a transplantation of gut microbiota from healthy donors to patients via the upper or lower part of the gastrointestinal tract. FMT may be administered as a fresh, frozen, lyophilized, or capsule-based formulation. 

**Figure 1:**
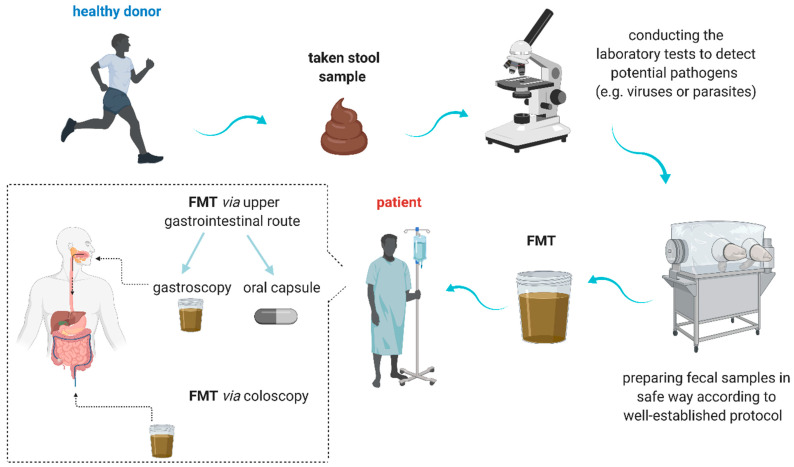
Fecal gut microbiota transplantation procedure [1,9,51,52]. FMT—fecal microbiota transplantation.

Firstly, the fecal samples collected from healthy subjects are screened to detect potential pathogens, such as viruses or parasites, afterward samples are prepared according to well-established procedure before transplantation (Figure 1 and Table 1) [1,9,51]. 

It should be noted that the efficacy and safety-aspects depend on the route of FMT and the quality of the content of stool samples taken from donors. Notwithstanding, the most beneficial route for FMT has not been established yet [53]. Below aforementioned aspects are discussed. Regarding administration routes, Kao et al. assessed whether FMT by oral capsule administration is not inferior to colonoscopy delivery in recurrent CDI [11]. This study included 116 participants divided into two groups: first group receiving FMT via colonoscopy (n = 59) and second consuming oral capsule (n = 57). It was noted that the rates of minor adverse events were higher in colonoscopy groups compared to patients receiving capsule (12.5% vs. 5.4%, respectively). Additionally, oral administration of FMT was not inferior to delivery via colonoscopy for preventing RCDI over 12 weeks. Therefore, FMT via oral capsule may be much more effective in treatment of RCDI compared to colonoscopy linked implementation [11]. Similarly, the efficiency of oral administration of frozen FMT capsules to treat RCDI was also investigated by Youngster et al. that included 180 patients with a large range of age (7–95 years) [54]. The analysis was conducted with a minimum follow-up period of 8 weeks after last oral FMT capsule administration. It was observed that in 82% of cases, CDI was resolved after one treatment with FMT. Moreover, this rate was higher (91%) if patients were treated with two FMT interventions [54]. Finally, a meta-analysis showed that frozen FMT is effective in treatment of RCDI as fresh FMT procedure (first effective rate 65.0% (95% CI 57.0–73.0%) vs. 65.0% (95% CI 57.0–73.0%), *p* = 0.962, respectively; pooled second effective rate 95.0% (95% CI 91.0–99.0%) vs. 95.0% (95% CI 92.0–99.0%), *p* = 0.880, respectively) [55]. Additionally, Luo et al. reported that administration of FMT via both oral capsules and colonoscopy are cost-effective strategies to treat RCDI [56]. A recently published systematic review and meta-analysis showed that oral FMT capsules are safe, as well as effective, for the treatment of RCDI [57]. 

### 3.2. FMT to Treat Multidrug-Resistant Bacteria Infections

The prevalence of multidrug-resistant bacteria (MDRB) is a growing problem worldwide [58], as a result of the inappropriate use of antibiotics [59]. Notably, bacteria should be considered as a MDR if it is resistant to at least one agent in ≥3 antibiotic classes (in which these bacteria are known to be susceptible) [60]. Patients with hematological malignancies are treated with broad spectrum antibiotics and multiple chemotherapeutic agents, thus with high risk of altered gut microbiota composition and consequently gut colonization of these bacteria [58]. Currently, the effective therapeutic strategies used to treat MDRB infections are limited. Nevertheless, FMT is a promising method [61], and it was shown that FMT successfully inhibited the *Klebsiella pneumoniae* MBL+ and *Escherichia coli* ESBL+ gut colonization in immunocompromised patients [62]. 

In a retrospective study, the efficacy of FMT before or after allo-HSCT in decolonization of MDRB was evaluated [60]. Ten participants (in four cases, FMT was given before allo-HSCT, and in six cases, it was given after this procedure) were enrolled in the study. Patients treated with FMT before allo-HSCT were colonized by carbapenemase-producing Enterobacteriaceae (CBPE) (n = 2) and carbenemase-producing *Pseudomonas aeruginosa* (n = 2). Subjects who received FMT after allo-HSCT were colonized by CBPE (n = 2), carbenemase-producing *Pseudomonas aeruginosa* (n = 2), and vancomycin-resistant enterococci (VRE) (n = 2). Complete decolonization was experienced by 7 patients (with median follow-up of 13 months after FMT). Moreover, all patients (n = 4) treated with FMT before allo-HSCT achieved persistent decolonization. Overall, FMT was presented as a safe method and adverse events included one case of constipation, one suffering from grade I diarrhea, and one having GvHD [60]. 

Antibiotic resistance is strongly associated with increased morbidity and mortality [12]. It is estimated that chronic infections with MDRB in patients who underwent allo-HSCT is related to a mortality rate raging between 36 and 95% [62,63,64,65]. In a single-center and prospective study, it was assessed whether FMT is effective in eradication of MDRB in patients (n = 20) with blood disorders (i.e., acute myeloblastic leukemia [n = 5], acute GvHD [n = 5], chronic GvHD [n = 2], multiple myeloma [n = 2], diffuse large B-cell lymphoma [n = 2], myelodysplastic syndrome [n = 1], lung cancer [n = 1], thrombotic thrombocytopenic purpura [n = 1], and kidney transplant recipient [n = 1]) [62]. Specifically, patients were colonized by the following MDRB: Klebsiella pneumoniae NDM1+ (n = 14), carbapenem-resistant *Klebsiella pneumoniae* (n = 3), *Klebsiella pneumoniae* extended-spectrum β-lactamase positive ESBL+ (n = 2), *Escherichia coli* ESBL+ (n = 11), *Pseudomonas aeruginosa* metallo-β-lactamase MBL (n = 2), carbapenem-resistant *Pseudomonas aeruginosa* (n = 2), carbapenem-resistant *Enterobacter cloacae* (n = 2), vancomycin-resistant enterococci (n = 2), and other strains of ARB (n = 3). A complete decolonization of ARB was noted in the 75% of patients whereas a partial decolonization in the 80% of participants. It was confirmed that FMT is safe and effective in the treatment of infections with ARB occurrence [62]. Recently, a systematic review and meta-analysis including five studies and 52 patients assessed the efficiency of FMT in eradication of MDRB [12]. It was shown that the decolonization of MDRB was achieved in half of the participants one month after FMT. Moreover, in the 70% of cases, the decolonization occurred within the first week after FMT [12]. These results confirmed that FMT may be an effective method used to treat infections with MDRB. Nevertheless, it is necessary to conduct further well-designed randomized clinical trials with an appropriate large sample size thus statistical power. 

Moreover, DeFillip et al. assessed whether third-part FMT following allo-HSCT had the potential to reconstitute the diversity of gut microbiota [66]. Overall, 18 patients were enrolled, but five were excluded due to development of acute gastrointestinal GvHD before FMT procedure (n = 3), persistent HCT-associated gastrointestinal toxicity (n = 1), and patient withdrawn (n = 1). Therefore, 13 patients received FMT capsules with a mean of 27 days after HCT (19–45 days). It was noted that two participants developed gastrointestinal GvHD (one patient also with bacteremia). Nevertheless, the restoration of gut microbiota diversity was observed after FMT, and the Kaplan–Meier estimate for 12-month OS was 85% (95% CI, 51–91%). These results confirmed that empiric third-party FMT after allo-HCT seems to be feasible and safe [66].

### 3.3. FMT and Clostridioides difficile Infection 

*Clostridioides difficile* belongs to anaerobic, Gram-positive, spore-forming bacillus [67]. CDI is strongly associated with prolonged hospitalization, increasing healthcare costs, as well as morbidity and mortality [68]. Immunosuppressed patients including patients with malignancies and/or HSCT recipients are at high risk of CDI development due to immune suppression and frequent use of antibiotics [67,68]. It is estimated that the incidence of CDI in these subjects is nine-fold higher than in general patients [69]. Therefore, there is a strong need for new therapeutic strategies to treat CDI and FMT seems to be a promising option. 

In a study by Webb and colleagues, seven HSCT recipients were treated with FMT predominantly (n = 6) via naso-jejunal route [70]. Overall, the 74.1% of patients (n = 5) were receiving immunosuppressive therapy. Mean follow-up was 265 days. Notably, no adverse events were experienced, and the mortality was 0%, demonstrating that with careful donor selection and laboratory screening, FMT could be a safe and effective therapy for CDI in HSCT patients [69]. In a single institution retrospective case series, the safety and efficiency of FMT for RCDI in patients with cancers treated with cytotoxic chemotherapy was evaluated [71]. This study included 23 participants (i.e., 13 patients with underlying hematologic and 10 with solid malignancies). Patients experienced a median of four CDI episodes and they had been treated before FMT (median 106 days) with the following antibiotics: vancomycin, metronidazole, or vidaxomycin. It was demonstrated that diarrhea was resolved (without recurrence) within 60 days after FMT in all patients, with negative *C. difficile* outcome. Two patients (9%) developed RCDI after 14 and 22 months from FMT. One patient died at day 5 after FMT due to cardiac arrest; however, the event was not related to FMT procedure. Overall, considering the 22 participants alive for 60 days or more after FMT, 48% (n = 11) underwent further chemotherapy and 43% (n = 9) received more antibiotics. These results confirmed that FMT is safe and strongly effective in the treatment of RCDI in patients receiving cytotoxic chemotherapy [71]. 

### 3.4. FMT and Graft-Versus-Host Disease 

Patients undergoing HSCT procedure may develop GvHD in the 40–80% of HSCT recipients [72,73] when alloreactive T cells from the donor are activated against healthy tissue in the recipients; however, the pathophysiology of acute GvHD (aGvHD) is not completely known [72]. Recently, Bilinski et al., in a case report, showed the effect of FMT in a 36-year-old man suffering from acute myelogenous leukemia [74]. FMT was used to decolonize gastrointestinal tract from ARB before allo-HSCT procedure; however, it caused transmission of norovirus and possibly induced eosinophilic gastroenteritis and GvHD. Nevertheless, these symptoms were resolved after the administration of steroids, as well as second FMT (norovirus-free) from another donor [74]. 

Interestingly, in a study including three cases after allo-HSCT, the results of FMT in the treatment of refractory gastrointestinal aGvHD were reported [75]. Overall, FMT was repeated from one to six times, and no bacteremia or systemic infection occurred immediately after FMT. Nevertheless, ten days after FMT, one patient developed bacteremia and 9 days after FMT, a patient died due to respiratory failure without bacteremia. After 77 days from the last FMT, another patient died due to septicemia not associated with FMT. Notwithstanding, the authors demonstrated that FMT seems to be a novel therapeutic approach for patients with refractory gastrointestinal GvHD [75]. In a pilot study including four patients with steroid-resistant (n = 3) and steroid-dependent (n = 1) acute intestinal GvHD, it was shown that FMT might be safe and useful as a new treatment option for these patients [76]. Notably, all patients responded to FMT and not related severe adverse events to this procedure were observed [76]. Similar results were obtained in other studies confirming that FMT could be a new treatment option for patients with intestine acute GvHD [77,78,79]. Additionally, the safety and efficacy of FMT for grade IV steroid refractory gastrointestinal tract GvHD was recently investigated by Zhao and colleagues [80]. This study included 55 patients (final statistics regarding 41 participants: n = 23 receiving FMT and n = 18 – control subjects). It was observed that the clinical remission was significantly greater in the group treated with FMT compared to the control group (on days 14 and 21 after FMT procedure). Moreover, an additional follow-up period of 90 days showed that the overall survival was better in FMT recipient. There was no difference in occurrence side effects between both groups [80]. DeFilipp et al. reported that drug-resistant *Escherichia coli* bacteremia occurred in two patients undergoing FMT and one of these patients died [81]. Therefore, it is recommended to enhance donor screening to reduce the risk of transmission of pathogens; thus, the safety of FMT needs further evaluation [80,81]. 

The cost-effectiveness aspects related to FMT were analyzed in some studies. For instance, Shaffer et al. assessed the cost-effectiveness of establishing FMT unit in Canada for the treatment of RCDI [82]. The authors reported that FMT is cost-effective in the aforementioned country (a sufficient number of eligible patients, i.e., 15–47, depending on the FMT modality used) [82]. Similarly, Varier et al. showed that FMT may be assessed as a cost-saving treatment of RCDI [83]. These researchers noted that FMT procedures cost less in comparison with vancomycin usage ($1669 and $3788, respectively). Other data also indicate that FMT is the most cost-effective method to treat RCDI [84]. 

The future perspective of gut microbiota modulations mainly via FMT in patients with hematologic diseases regarding selected recruiting and not yet recruiting trials registered in ClinicalTrials.gov (accessed on 12 February 2021) are presented in Table 2. 

## 4. Role of Nutritional Interventions, Probiotics, Prebiotics, Synbiotic, and Postbiotics in Hematologic Patients in the Context of Gut Microbiota Modulation 

### 4.1. Nutritional Interventions 

The modulation of gut microbiota through nutritional interventions, such as enteral and parenteral nutrition exists [85]. Recently, it was shown that enteral nutrition promotes the recovery of gut microbiome homeostasis in children undergoing allo-HSCT [86]. The restoration of gut microbiota after HSCT procedure may reduce the risk of GvHD and systemic infection [86]. Some studies revealed that parenteral nutrition is related to loss of commensal bacteria (regarding genus Blautia), promotes bacterial translocation, and alters the production of short-chain fatty acids [31,87]. Nevertheless, not only enteral/parenteral nutrition affects gut microbiota in HSCT recipients, but also particular nutrients. For instance, lactose, a common element of nutrition may exacerbate intestine and systemic inflammatory diseases [88]. In their study, Stein-Thoeringer et al. assessed the role of Enterococci in aGvHD development in allo-HSCT patients (n = 1325), as well as in preclinical allo-HSCT mouse models [88]. The fecal microbiota was investigated by using 16S rRNA gene sequencing. The authors reported that the growth of Enterococcus depends on disaccharide lactose. The depletion of dietary lactose attenuates the outgrowth of Enterococcus and then decreases the severity of GvHD in mouse model [88]. 

### 4.2. Probiotics 

According to the Food and Agriculture Organization of the United Nations and World Health Organization (FAO/WHO), probiotics are defined as “live microorganisms which when administered in adequate amounts confer a health benefit on the host” [1,89]. Among them, *Lactobacillus* spp. and *Bifidobacterium* spp. are the most commonly used probiotic microorganisms [90]. The survey conducted in single center showed that 28.5% of 499 cancer patients declared probiotic usage [91,92]. 

Chemotherapeutic treatments may cause development of gastrointestinal adverse events, such as mucositis and deficient absorption of several nutrients [5]. Therefore, the reduction of these adverse events is strongly needed. In an animal model study (C57BL/6 and B6D2F1 mice), it was shown that the administration of *Lacticaseibacillus rhamnosus* GG orally before and after HSCT reduced significantly aGvHD score and improved the survival rate [93]. Nevertheless, in a subsequent study including 80 patients who underwent HSCT, *L. rhamnosus* GG reduced the risk of stage III-IV of aGvHD but have no impact on overall incidence of GvHD [94]. Moreover, a randomized pilot study, assessed whether supplementation with *L. rhamnosus* can be effective in chemotherapy-induced gastrointestinal side effects reduction in patients with acute leukemia [95]. The participants were randomized into two groups: the first receiving 5 × 10^9^ CFU probiotic orally twice a day and the second as control without probiotics administration. The gastrointestinal side effects, such as vomiting, nausea, and abdominal distension were significantly decreased in the probiotic group (*p* < 0.05) [95]. In addition, it was revealed that one of the antibiotics, i.e., irinotecan actively used during allo-HSCT, may be influenced by commensal bacteria which converts this drug to active metabolite SN-38 by beta-glucuronidase, indicating epithelial barrier damage and mucositis and subsequently worsening of diarrhea. What is worth pointing is that the administration of probiotics in colon cancer treated with irinotecan, indicated significant reduction of severity and incidence of the gastrointestinal toxicity [91,96]. 

In an acute leukemia mouse model, the restoration of *Lactobacillus* species (in particular *Limosilactobacillus reuteri* 100-23 and *L. gasseri* 311476) in the gut microbiota was related to a decreased inflammation, reduction of inflammatory factors (i.e., interleukin-6, monocyte chemoattractant protein-1, interleukin-4, and granulocyte colony-stimulating factor) and a reduced expression of muscle atrophy markers (i.e., Atrogin-1, MuRF1, LC3, and Cathepsin L) [97]. 

Despite the abovementioned potential benefits of probiotic agents, their safety and tolerability in patients with hematologic malignancies is still controversial. Recently, Koyama et al. presented a case of septicemia due to *L. rhamnosus* GG (using a probiotic-enriched yogurt) in a 54-year-old male with acute promyelocytic leukemia who was autologous HSCT (auto-HSCT) recipient [98]. Due to severe diarrhea, he received probiotic-enriched yogurt, and one week later, he developed a septic shock. It should be emphasized that the pathogen was determined by strain-specific PCR analysis as *L. rhamnosus* GG (ATCC 53103), and it was identical as the strain found in consumed yogurt. On the other hand, there are also studies confirming that administration of probiotics is safe for patients undergoing HSCT [99,100]. Nevertheless, it should be emphasized that the properties of probiotics are strongly species-dependent and probiotic strains should be given carefully to HSCT recipients [98]. 

*Saccharomyces boulardii* CNCM I-745 is a non-bacterial microorganism belonging to *Saccharomyces*, which may be used as a probiotic agent in supportive treatment of antibiotic-associated diarrhea, *Helicobacter pylori* infection, candidiasis, and others [101]. However, the use of fungal probiotics in onco-hematological patients is still controversial, and no guidelines on the routine *S. boulardii* ingestion in these patients have been published so far [2]. Notably, it is estimated that, around 90% of patients being prepared for HSCT suffer from oral mucositis [102], which may contribute to yeast translocation through oral mucus membrane into the bloodstream and lead to severe infection [2] with cases on *S. boulardii* and *S. cerevisiae* and sepsis reported [103,104,105]. Nevertheless, a retrospective analysis demonstrated that, despite the colonization of many onco-hematological patients with *Saccharomyces* spp., cases of fungal sepsis were not observed [106]. 

### 4.3. Prebiotics 

Prebiotics are defined as selectively fermentable, non-digestible oligosaccharides or ingredients which alter the composition and activity of gut microbiota conferring health benefits [107]. In a retrospective study, Iyama and colleagues assessed whether enteral nutrition enriched with three components, such as glutamine, fiber, and oligosaccharide (GFO), is effective in decreasing mucosal damage in HSCT recipients [108]. This study included 44 participants divided into two groups, a group receiving GFO and a control group without supplementation. Two packages (one package containing: 36 kcal, 3 g of glutamine, 5 g of dietary fiber, 1.5 g of oligosaccharide, and 1.2 mg of sodium) of GFO dissolved in 200 mL of water were given to patients orally three times per day (beginning 7 days prior to the start of conditioning and continued until 28 days after HSCT). Grade 3/4 diarrhea was lasted shorter in the GFO group in comparison to control subjects (0.86 vs. 3.27 days, respectively). Similarly, incidence of grade 3/4 mucositis was reduced in the GFO group compared to control (3.86 vs. 6), and survival rate at 100 days was 77.3% in control group and 100% in participants receiving GFO. Overall, this supplementation may be effective as supportive treatment of mucosal injury in these patients [108]. 

### 4.4. Synbiotics 

Synbiotics are described as a combination of probiotic bacteria and growth-promoting prebiotic ingredients that achieve “synergism” [107]. The data regarding the use of synbiotics in onco-hematological patients are very limited. In an animal model study with leukemic mice with cachexia, it was shown that a synbiotics contained inulin-type fructan and *L. reuteri* 100-23 restored intestinal homeostasis and prolonged survival [109]. After the administration of a synbiotic, the restoration of *Lactobacillus* species and the reduction of *Enterobacteriaceae* counts were observed. Additionally, the decreases of hepatic cancer cell proliferation, muscle wasting, and morbidity, as well as prolonged survival, were reported. 

### 4.5. Postbiotics 

Nowadays, there is observed growing attention towards postbiotics in the context of gut microbiota modulation being strongly associated with the production and secretion of multiple metabolites. Postbiotics are functional bioactive compounds that do not contain any organisms, mainly constituted by exopolysaccharides, supernatant, cellular wall fragment, cell lysates, teichoic acid, peptidoglycan-derived muropeptides, SCFAs, vitamins, and phenols [110,111]. There is high structural heterogeneity of postbiotics, and thus various techniques are used to their acquisition. Postbiotics which are non-live bacterial products can be treated as an attractive alternative for immunosuppressed patients who have higher risk of infection after administration of live bacteria [112]. There is a wide diversity of microbial products which induce different processes in organism. For instance, butyrate acid inhibits lymphocyte proliferation or IL-2 production and has anti-inflammatory effect on intestinal epithelial and immune cells [113,114]. In addition, SCFAs can also inhibit hematopoiesis or increase T-regs’ level in lungs, preventing lung allergic inflammation [115,116]. Due to many interactions between microbe-associated molecular patterns (MAMPS) and both the innate immune cells and non-immune cells of the host, the impact of postbiotics allo-HCST should be investigated but only few studies on the topic have been published so far. Current findings suggest that microbial metabolites can have a significant issue in patients undergoing allo-HSCT [117]. Indeed, microbe-derived SCFAs, such as butyrate and propionate, in systematic circulation are related to protection from GVHD occurring after allo-HSCT [118]. Other studies also emphasized that 17 rationally selected strains of *Clostridia* (producing the SCFA butyrate) increase T-regs in the gut [119]. Moreover, an in vitro study reported that systemic administration of tauoro-urso-deoxycholic acid decreased GVHD severity in three different murine transplantation models, an effect mediated by the decreased activity of the antigen presentation machinery and prevention of apoptosis of the intestinal epithelium. However, administration of this bile acid did not change bacterial composition in intestinal epithelium, suggesting therefore a cell-specific instead of a microbiome-related mechanism [120]. Metabolomics analysis of acute GVHD onset has demonstrated that HCST is followed by major changes in metabolomics profiles of recipients. Metabolomics changes in microbiota-derived metabolites were observed in patients who developed acute GVHD in comparison to healthy subjects with a decreased production of aryl hydrocarbon receptor ligands and plasmalogens and increased production of bile acids. These changes can limit indoleamine 2,3-dioxygenase induction and influence allogenic T-cell reactivity [121]. Finally, the role of bile acids is unclear, with data suggesting an involvement in enhancing pro-inflammatory cytokines production, T-cell activation, and neutrophil recruitment [122]. On the other hand, other data indicate that bile acids can inhibit inflammasome activation [123].

## 5. Conclusions 

Gut microbiota can have a significant role in the management of hematologic malignancies, and several studies have been performed in the last years. Modifications of its composition and activity seem to be a therapeutic option in patients suffering from these disorders. Currently, FMT is the most modern method used to modulate and restore gut microbiota balance, but its use in hematologic disorders is still limited. The available data assessing FMT efficiency most often regard small sample size, and despite some adverse events occurring after FMT, the side effects directly related to this procedure rarely occur. Finally, the administration of prebiotics, probiotics, and synbiotics as a therapeutic strategy to modulate gut microbiota and support standard treatment is promising. Nevertheless, the administration should be considered carefully due to the high risk of fungemia/septicemia. 

## Figures and Tables

**Table 1:** Most common preliminary tests for FMT [51].

The Most Common Preliminary Tests for FMT	
Bacterial serology	Treponema palladium
Viral serology	Hepatitis A virus IgM, hepatitis B surface antigen, hepatitis C antibody, cytomegalovirus, and Epstein–Barr virus
Parasite serology	*Strongyloides stercoralis* and *Entamoeba histolytica*
Blood tests	Complete blood count, complete metabolic panel, liver tests (i.e., aspartate aminotransferase, alanine aminotransferase, alkaline phosphatase, total bilirubin, and C-reactive protein)
Stool tests	Stool *Clostridium difficile* studies (toxin polymerase chain reaction (PCR), enzyme-linked immunoassay (ELISA), and toxigenic culture)
Bacterial stool tests	*Salmonella*, *Shigella*, *Campylobacter* cultures, *E. coli* O157 culture, *H. pylori* immunoassay, and vancomycin-resistant *Enterococcus* culture
Viral stool tests	Adenovirus ELISA, norovirus ELISA or quantitative PCR, and rotavirus ELISA
Parasite stool tests	Ova and parasite microscopy, Microsporidia microscopy, Giardia fecal antigen ELISA, Cryptosporidium ELISA, and Isospora and Cyclospora microscopy

**Table 2:** Future perspective of therapeutic modification of gut microbiota in patients with hematologic diseases. Selected recruiting/not yet recruiting trials registered in ClinicalTrials.gov (accessed on 12 February 2021).

Identifier	Title of the Study	Study Type	Disease/ Condition	Sample Size (n)	Interventions/ Treatment	Primary Outcomes	Current Status
NCT03922035	“CBM588 in improving clinical outcomes in patients who have undergone donor hematopoietic stem cell transplant”	Pilot study	Hematopoietic and lymphoid cell neoplasm	36	*Clostridium butyricum* CBM 588 probiotic strain	Adverse events	Recruiting
NCT04269850	“Fecal microbiota transplantation with ruxolitinib and steroids as an upfront treatment of severe acute intestinal GVHD”	Pilot study	Intestinal GVHD	20	Allogenic FMT	Overall survival	Recruiting
NCT03678493	“A study of FMT in patients with AML allo HSCT in recipients”	Randomized placebo- controlled trial	AML, ASCT	120	FMT	Incidence of infections	Recruiting
NCT03819803	“Fecal microbiota transplantation in aGvHD after ASCT”	Interventional	GvHD in GI Tract	15	FMT	GI-aGvHD remission	Recruiting
NCT04593368	“Faecal microbiome transplantation (FMT) in pediatric patients colonized with antibiotic-resistant pathogens before hematopoietic stem cell transplantation (HSCT)”	Prospective non-randomized phase II trial	Pediatric patients colonized with antibiotic-resistant pathogens before HSCT	15	oral dosing of fecal microbiome from allogeneic donor	Frequency of decolonization	Not yet recruiting
